# MYC2 Transcription Factors TwMYC2a and TwMYC2b Negatively Regulate Triptolide Biosynthesis in *Tripterygium wilfordii* Hairy Roots

**DOI:** 10.3390/plants10040679

**Published:** 2021-04-01

**Authors:** Yanbo Huo, Jing Zhang, Bin Zhang, Ling Chen, Xing Zhang, Chuanshu Zhu

**Affiliations:** 1 College of Plant Protection, Northwest A&F University, Yangling 712100, China; huoyanbo@nwsuaf.edu.cn (Y.H.); zhjing008@nwsuaf.edu.cn (J.Z.); zhangbin1990@nwsuaf.edu.cn (B.Z.); Chenling@nwsuaf.edu.cn (L.C.); 2 Shaanxi Research Center of Biopesticide Engineering & Technology, Northwest A&F University, Yangling 712100, China

**Keywords:** MYC2, bHLH, transcriptional regulation, triptolide, RNA interference, metabolic engineering

## Abstract

Triptolide, an important bioactive diterpenoid extracted from the plant *Tripterygium wilfordii*, exhibits many pharmacological activities. MYC2 transcription factor (TF) plays an important role in the regulation of various secondary metabolites in plants. However, whether MYC2 TF could regulate the biosynthesis of triptolide in *T. wilfordii* is still unknown. In this study, two homologous MYC2 TF genes, *TwMYC2a* and *TwMYC2b*, were isolated from *T. wilfordii* hairy roots and functionally characterized. The analyses of the phylogenetic tree and subcellular localization showed that they were grouped into the IIIe clade of the bHLH superfamily with other functional MYC2 proteins and localized in the nucleus. Furthermore, yeast one-hybrid and GUS transactivation assays suggested that TwMYC2a and TwMYC2b inhibited the promoter activity of the miltiradiene synthase genes, *TwTPS27a* and *TwTPS27b*, by binding to the E-box (CACATG) and T/G-box (CACGTT) motifs in their promoters. Transgenic results revealed that RNA interference of *TwMYC2a/b* significantly enhanced the triptolide accumulation in hairy roots and liquid medium by upregulating the expression of several key biosynthetic genes, including *TwMS* (*TwTPS27a/b*), *TwCPS* (*TwTPS7/9*), *TwDXR*, and *TwHMGR1*. In summary, our findings show that TwMYC2a and TwMYC2b act as two negative regulators of triptolide biosynthesis in *T. wilfordii* hairy roots and also provide new insights on metabolic engineering of triptolide in the future.

## 1. Introduction

*Tripterygium wilfordii*, common name Lei Gong Teng or thunder duke vine, has a long history in traditional Chinese medicine, mainly to treat rheumatoid arthritis [1,2]. The identification of hundreds of effective compounds isolated from the roots of *T wilfordii*, such as diterpenoids, triterpenoids, and sesquiterpenoids [3,4], has stimulated the study of their pharmacological properties. In *T. wilfordii*, much of the observed pharmacological activity can be attributed to the presence of triptolide, which has a variety of pharmacological activities, such as antitumor, anticancer, immunosuppressive, and anti-inflammatory activities [5,6,7,8]. Recently, numerous studies have focused on investigating the biosynthetic pathway of triptolide, leading to the isolation and functional characterization of many triptolide biosynthetic pathway genes, including not only the upstream pathway genes, such as *TwHMGR1* [9], *TwHMGS* [10,11], *TwDXR* [12,13], *TwDXS1/2* [12,14], *TwHDR* [15], *TwIDI* [16,17], and *TwGGPPS1/4/8* [18,19], but also the downstream pathway genes, such as *TwTPS7/7v2/9/9v2* [20,21], *TwTPS27/27v2* [20,21], and *TwCYP728B70* [22] (all of these functional pathway genes’ full names are shown in Table S1). However, less is known about the transcriptional regulation of triptolide pathway in *T. wilfordii*, as compared with the progress of triptolide biosynthetic genes. 

MYC2 transcription factor (TF), which belongs to the basic helix-loop-helix (bHLH) superfamily, has been shown to involve in the regulation of plant secondary metabolism. In *Arabidopsis thaliana*, AtMYC2 enhances the production of sesquiterpene, especially (*E*)-β-caryophyllene, by upregulating the sesquiterpene synthase genes *TPS21* and *TPS11* [23]. In *Chimonanthus praecox*, overexpression of CpMYC2 in model plant *A. thaliana* shows an increase in the production of monoterpene linalool [24]. In *Salvia miltiorrhiza*, SmMYC2a positively regulates the biosynthesis of diterpenoid tanshinone by affecting multiple pathway genes in tanshinone biosynthesis [25]. In *Withania somnifera*, WsMYC2 upregulates the biosynthesis of triterpenoid withanolides via key pathway genes *WsCYP85A* and *WsCYP90B* [26]. These findings also suggest that MYC2 promotes a wider range of terpenoids biosynthesis. In addition to the positive effects of MYC2, the negative role of MYC2 in regulating plant secondary metabolism has also been reported but limited. For example, AtMYC2 negatively controls Trp and Trp-derived indole glucosinolate biosynthesis during JA signaling in *A. thaliana* [27]. TcJAMYC1/2/4, three homologous of AtMYC2, display a negative role in taxol biosynthetic gene expression, at least when transiently overexpressed in *Taxus cuspidata* cultured cells [28]. However, whether MYC2 could up- or down-regulate the diterpenoid triptolide biosynthesis in *T. wilfordii* is still unknown. Therefore, it is necessary to isolate the MYC2 TF gene from *T. wilfordii* and identify its potential function in triptolide biosynthesis. 

It has been reported that the E-box elements ‘CANNTG’ and its variants ‘CACGTG’ (called G-box) and ‘CACGTT’ (called T/G-box) in the promoters of pathway genes can be bound by bHLH and MYC2 TFs which are involved in plant secondary metabolism [29,30,31,32]. For example, SmbHLH10 enhances the tanshinone biosynthesis in *S. miltiorrhiza* by directly binding to the T/G-box (CACGTT) and G-box like (CACGTC) motifs in the promoters of key enzyme genes *SmCPS1*, *SmCPS5*, and *SmDXS2* [33]. AsMYC2 upregulates agarwood sesquiterpene biosynthesis by directly binding to the G-box motif in the promoter of *ASS1*, a key sesquiterpene synthase gene in *Aquilaria sinensis* [34]. These findings suggest that the E-box motifs in the key pathway gene promoters play important roles in the regulation of secondary metabolism in plants. Recently, in our previous studies, five different types of E-box elements, including CAGATG, CACATG, CAAATG, CACGTT (T/G-box), and CAATTG, were found in the promoters of miltiradiene synthase genes, *TwTPS27a* and *TwTPS27b*, key genes of triptolide biosynthesis in *T. wilfordii* [35]. This indicated that these E-box elements in the promoters of *TwTPS27a/b* as the potential MYC binding sites might be recognized by MYC2 TF in *T. wilfordii*. Therefore, the interaction between the *TwTPS27a/b* promoters and MYC2 TF that may regulate triptolide biosynthesis needs further investigation. 

RNA interference (RNAi) technology is often used for the functional identification of candidate genes in vivo in plants [36]. *Agrobacterium rhizogenes*-mediated RNA interference (RNAi) technology has been successfully used to identify transcription factors (TFs) function and regulate the biosynthesis of secondary metabolites in many plants, such as *Salvia miltiorrhiza* [25], *Catharanthus roseus* [37], *Nicotiana benthamiana* [38], *Coptis japonica* [39], and *Ophiorrhiza pumila* [40]. For example, RNA interference-mediated knockdown experiments with hairy roots of *Salvia miltiorrhiza* suggested that SmMYC2a is a positive regulator in the biosynthesis of tanshinones [25]. Artificial micro RNA mediated suppression of WsMYC2 was performed in *W. somnifera* leaves to confirm its positive role in regulating withanolide biosynthesis by inhibiting key pathway genes [26]. Recently, our lab established a rapid and efficient *Agrobacterium rhizogenes*-mediated RNAi system, generating stable transgenic hairy roots of *T. wilfordii* [9]. Therefore, the establishment of a transgenic RNAi hairy roots system will contribute to identifying the function of candidate TFs that may be involved in the regulating of triptolide biosynthesis.

To date, no MYC2 TF gene has been isolated and characterized by its function in *T. wilfordii*. The purpose of this study is to identify and find out the potential role of the MYC2 in regulating triptolide biosynthesis of *T. wilfordii*. We isolated two homologous MYC2 TFs named TwMYC2a and TwMYC2b. They are homologous with other plant MYC2 proteins and phylogenetic analysis showed that they were grouped into the IIIe clade of the bHLH superfamily with other functional MYC2 proteins. The subcellular localization analysis implied that TwMYC2a/b are two nuclear-localized proteins. The yeast one-hybrid (Y1H) and GUS transactivation assays confirmed the interaction between the TwMYC2a/b proteins and the promoters of miltiradiene synthase genes, *TwTPS27a* and *TwTPS27b*, which are the key genes in triptolide biosynthesis. Furthermore, *TwMYC2a/b* were silenced using RNA interference (RNAi) in *T. wilfordii* hairy roots. The expression of several key pathway genes of triptolide biosynthesis and production of triptolide were analyzed in the *TwMYC2a/b*-RNAi and control hairy roots lines. Taken together, our results indicate that TwMYC2a and TwMYC2b are two negative regulators of triptolide biosynthesis in the hairy roots of *T. wilfordii* and provide new insights on metabolic engineering of triptolide in the future. 

## 2. Results

### 2.1. Isolation and Molecular Characterization of TwMYC2a and TwMYC2b

Through homolog searching, one candidate gene, *comp41180_c0_seq5*, was selected from the *T. wilfordii* transcriptome library (SRX472292) with the highest score (Figure S1). This candidate gene was homologous to the known transcription factor (TF) AtMYC2 (as the query protein) from *Arabidopsis thaliana*. To obtain the full-length cDNA of this candidate gene, the 5′/3′-rapid amplification of cDNA ends (5′/3′-RACE) and full-length coding sequence (CDS) cloning assays were performed. The identification of polymerase chain reaction (PCR) products of the candidate gene was shown in Figure S2. After sequence alignment (Figures S3 and S4), two homologous nucleotide sequences were obtained and named *TwMYC2a* and *TwMYC2b*, respectively. Then, the sequences of *TwMYC2a* and *TwMYC2b* including 5′/3′-untranslated region (5′/3′-UTR) and full-length CDS were submitted to the National Center for Biotechnology Information (NCBI) database and were given the Genbank accession numbers MN836714 and MN836715, respectively. 

**Figure 1:**
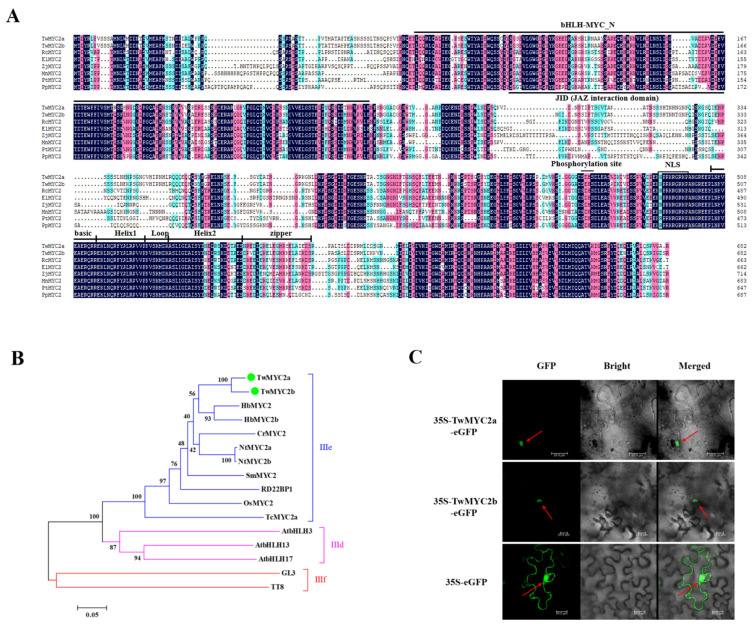
Multiple sequence alignment, phylogenetic analysis, and subcellular localization analyses of TwMYC2a/b. (**A**) Multiple sequence alignment of the TwMYC2a/b protein sequences with their homologous MYC2 proteins from different plant species: *Ricinus communis* RcMYC2 (XP_002519814.1), *Euphorbia lathyris* ElMYC2 (AVA18027.1), *Ziziphus jujuba* ZjMYC2 (XP_015896923.1), *Morus notabilis* MnMYC2 (XP_010104300.1), *Populus tomentosa* PtMYC2 (AZQ19203.1), and *Prunus persica* PpMYC2 (XP_020419289.1). The bHLH-MYC-N domain, bHLH-leucine zipper domain, and phosphorylation site were marked with an overline. JAZ interaction domain was marked with an underline. The nuclear localization signal (NLS) was represented by a solid box. (**B**) Phylogenetic analysis of TwMYC2a/b and other clades IIId/IIIe/IIIf bHLHs. Numbers at nodes represent bootstrap values (based on 1000 resamplings). The scale bar indicates the evolutionary distance. TwMYC2a/b are represented by large green dots. Accession NO. in GenBank: AtMYC2 (also called RD22BP1, BAA25078), AtbHLH3 (AAL55710), AtbHLH13 (Q9LNJ5), AtbHLH17 (Q9ZPY8), GL3 (NP_193864), TT8 (OAO98324), TcMYC2a (ATY38591), OsMYC2 (0336P5), SmMYC2 (AO09733), NtMYC2a (ADU60100), NtNYC2b (ADU60101), CrMYC2 (AQ14332), HbMYC2 (AJC01627), and HbMYC2b (XP_021664832). (**C**) Subcellular localization analyses of the TwMYC2a/b proteins in tobacco (*Nicotiana benthamiana*). TwMYC2a/b fused in-frame with an enhanced green fluorescent protein (eGFP) was transiently expressed in tobacco leaf cells. The empty vector 35S::eGFP was used as a control. The red arrows indicate the location of the nucleus. Scale bars are 20 μm.

Based on the NCBI ORF finder, the CDSs of *TwMYC2a* and *TwMYC2b* are both 2049 bp, encoding 682 aa. The alignment of the full-length CDSs and protein sequences between *TwMYC2a* and *TwMYC2b* revealed that these two genes were homologous, sharing 93.67%/90.19% nucleotide/protein sequence identity (Figure S5). The theoretical isoelectric point (pI) and the molecular weight (Mw) of the TwMYC2a and TwMYC2b proteins were 5.19/74.43 kDa (pI/Mw) and 5.50/74.44 kDa (pI/Mw), respectively. Moreover, multiple sequence alignment showed that both the TwMYC2a/b proteins possess a bHLH-MYC superfamily domain at the N-terminal region and a basic helix-loop-helix leucine zipper domain at the C-terminal region (Figure 1A). Additionally, the 4aa-length peptides in the TwMYC2a/b proteins (‘SDHS’ for TwMYC2a and ‘SEHS’ for TwMYC2b, Figure 1A), assumed to be the phosphorylation sites, were only highly similar among MYC2 proteins [41]. Phylogenetic analysis revealed that they were grouped into the IIIe clade of the bHLH superfamily with other functional MYC2 proteins (Figure 1B), which were previously shown to regulate the biosynthesis of plant secondary metabolites [27,37,42,43,44,45,46], suggesting that TwMYC2a/b are likely to be involved in the regulation of secondary metabolism in *T. wilfordii*. Besides, both the TwMYC2a/b proteins also have a predicted typical nuclear localization signal (NLS) (Figure 1A). The subcellular localization analyses of the TwMYC2a/b proteins were performed in tobacco leaf cells (Figure 1C). As shown in Figure 1C, the fluorescence of GFP was detected in the cytoplasm and nucleus in the tobacco leaves transformed with the empty vector (35S-eGFP). In contrast to this, the fluorescent signals of 35S-TwMYC2a-eGFP and 35S-TwMYC2b-eGFP fusion proteins were only detected in the nucleus. These data implied that TwMYC2a and TwMYC2b are nuclear-localized proteins. 

### 2.2. TwMYC2a and TwMYC2b Specifically Bind the E-Box and T/G-Box Motifs within the TwTPS27a and TwTPS27b Promoters

**Figure 2:**
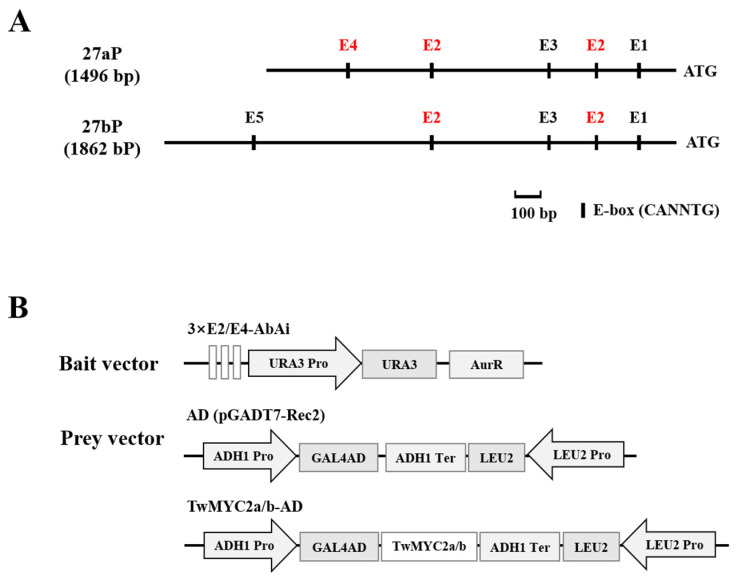

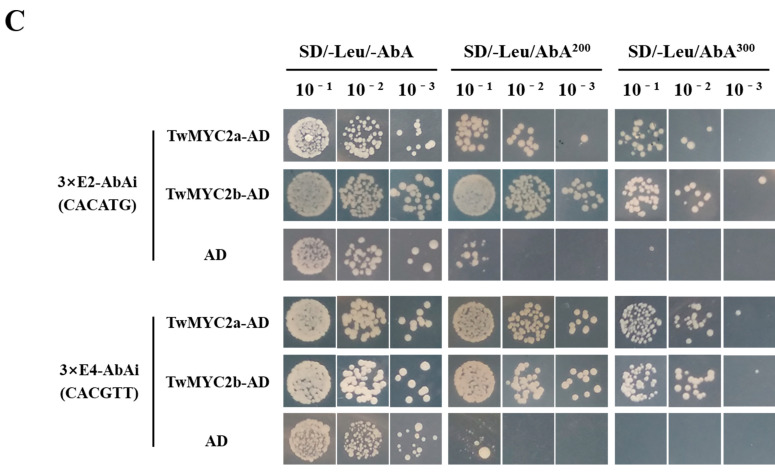
TwMYC2a/b could bind to the E-box (CACATG) and T/G-box (CACGTT) in yeast cells. (**A**) Schematic diagram of the different E-box motifs present in the *TwTPS27a* and *TwTPS27b* promoters. These motifs are E1 (CAGATG), E2 (CACATG), E3 (CAAATG), E4 (CACGTT, called T/G-box), and E5 (CAATTG), respectively. ATG, the start codon. (**B**) Schematic diagrams of the bait and prey vectors used for yeast one-hybrid (Y1H) assays. The triple repeated sequences of E-box (E2, CACATG) and T/G-box (E4, CACGTT) were synthesized and ligated into pAbAi, while the full-length coding sequences of *TwMYC2a* and *TwMYC2b* were separately fused with GAL4 AD in pGADT7-Rec2. (**C**) Y1H assays showing that TwMYC2a/b could bind to the E-box (E2, CACATG) and T/G-box (E4, CACGTT) present in the *TwTPS27a/b* promoters, based on the ability of the transformed Y1HGold yeast cells to grow on SD/−Leu/AbA^200^ and SD/−Leu/AbA^300^ medium in the gradient dilution (1/10, 1/100, 1/1000). The transformants grown on SD/−Leu/−AbA plate were used as positive controls for transformants growth. Positive transformants were confirmed by spotting yeast cells onto agar medium of SD/−Leu with 200 and 300 ng/mL AbA. These assays were repeated three times with similar results.

It is well known that MYC2 TFs recognize the E-box (CANNTG) and its variants G-box (CACGTG) or T/G-box (CACGTT) within the promoters of their target genes [47,48,49,50,51,52]. Our previous studies [35] showed that several different types of E-box motifs (CANNTG) were found in the promoters of miltiradiene synthase genes, *TwTPS27a* and *TwTPS27b*, which are the key genes in triptolide biosynthesis. As shown in Figure 2A, there are five different E-box motifs present in the *TwTPS27a* and *TwTPS27b* promoters, including E1 (CAGATG), E2 (CACATG), E3 (CAAATG), E4 (CACGTT, called T/G-box), and E5 (CAATTG), indicating that these motifs may be the binding sites of TwMYC2a and TwMYC2b.

To verify the binding of TwMYC2a/b to the E-box (E2, CACATG) and T/G-box (E4, CACGTT) motifs of *TwTPS27a/b* promoters, yeast one-hybrid (Y1H) assays were performed in Y1HGold yeast cells. After introducing the vacant pGADT7-Rec2 (AD) vector into the yeast cells harboring the bait vectors, 3×E2-AbAi or 3×E4-AbAi (Figure 2B), it was found that the growth of the transformed yeast cells was inhibited on an SD/−Leu plate under 200 and 300 ng/mL concentration of aureobasidin A (AbA), which is a potent and unique yeast antibiotic (Figure 2C). However, when transformed with the prey vectors TwMYC2a-AD/TwMYC2b-AD (Figure 2B), the yeast cells harboring 3×E2-AbAi or 3×E4-AbAi vectors were still able to grow normally on an SD/−Leu/AbA^300 ng/mL^ plate (Figure 2C). These findings suggested that TwMYC2a and TwMYC2b were indeed able to bind the E-box (CACATG) and T/G-box (CACGTT) present in the *TwTPS27a/b* promoters, which was consistent with the expectation of MYC2 TFs.

### 2.3. TwMYC2a and TwMYC2b Could Repress the TwTPS27a and TwTPS27b Promoters

**Figure 3:**
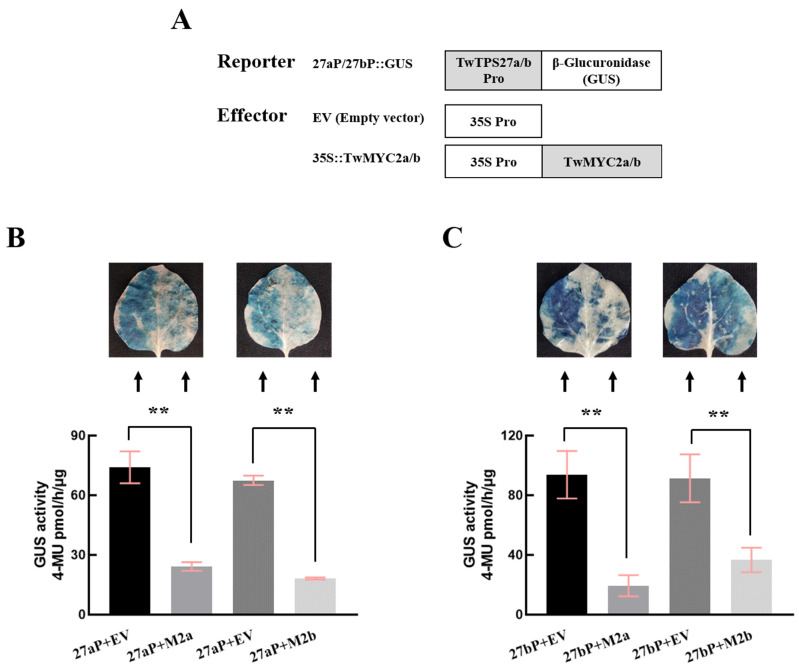
Repression of the *TwTPS27a* and *TwTPS27b* promoters by transient overexpression of *TwMYC2a* and *TwMYC2b* in tobacco leaves. (**A**) Sketch map of the reporter and effector vectors for GUS staining and GUS fluorimetric assays. 27aP::GUS and 27bP::GUS represent the promoter expression vectors constructed by introducing the promoter sequences of *TwTPS27a* (1496 bp) and *TwTPS27b* (1862 bp) into the pBI121 vector, respectively. Based on the GUS staining and GUS fluorimetric analysis of the infiltrated tobacco leaves, TwMYC2a and TwMYC2b significantly decreased the promoter activity of *TwTPS27a* (**B**) and *TwTPS27b* (**C**). Different combinations of the mixed bacterial suspension were infiltrated into the left and right sides of the same tobacco leaves, respectively. 27aP and 27bP represent the 27aP::GUS and 27bP::GUS. M2a and M2b represent the 35S::TwMYC2a and 35S::TwMYC2b. GUS activity of the infiltrated leaves was measured after infiltration for 72 h. The value of GUS activity was measured as pmol of 4-MU generated per hour per μg protein. All data presented here are means ± SD of three biological replicates. Statistical significance was determined by Student’s *t*-test (** *p* < 0.01). These assays were repeated three times with similar results.

To examine whether TwMYC2a and TwMYC2b directly regulate the promoters of *TwTPS27a* and *TwTPS27b* in tobacco (*N. benthamiana*) leaves, the effectors 35S::TwMYC2a/b or empty vector (EV) (Figure 3A) co-transformed with the reporters 27aP::GUS or 27bP::GUS (Figure 3A) into tobacco leaves, respectively, for β-glucuronidase (GUS) transactivation assays. As shown in Figure 3B,C, the GUS staining analysis showed that the staining intensity was lower on the right side of the midrib in the same infiltrated tobacco leaves, which were co-transformed with 27aP::GUS and 35S::TwMYC2a/35S::TwMYC2b, or 27bP::GUS and 35S::TwMYC2a/35S::TwMYC2b, than their corresponding controls, which were co-transformed with EV and 27aP::GUS, or EV and 27bP::GUS, on the left side of the midrib in the same infiltrated tobacco leaves. Similarly, their GUS fluorimetric activities also decreased significantly, compared with their corresponding controls (Figure 3B,C), indicating that the transient overexpression of *TwMYC2a* and *TwMYC2b* could strongly decrease the activity of the *TwTPS27a* and *TwTPS27b* promoters. Therefore, these results revealed that TwMYC2a and TwMYC2b both negatively regulated the activity of the *TwTPS27a* and *TwTPS27b* promoters, and also implied that TwMYC2a/b may play roles in down-regulating the biosynthesis of triptolide.

### 2.4. Relative Expression of TwMYC2a/b and Key Pathway Genes in the RNA Interference (RNAi) Hairy Roots of T. wilfordii 

**Figure 4:**
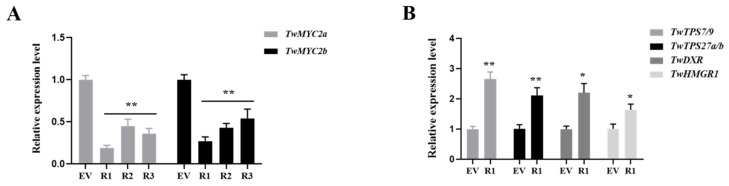
Relative expressions of *TwMYC2a*/*b* (**A**) and several key triptolide biosynthesis genes (**B**) in the RNAi transgenic and control hairy roots lines. Elongation factor 1 α (EF1α) was used as the internal standard for gene expression normalization. All data presented here are means ± SD of three biological replicates. Statistical significance was determined by Student’s *t*-test (* *p* < 0.05; ** *p* < 0.01). These assays were repeated two times with similar results.

To investigate the regulatory role of TwMYC2a/b in triptolide biosynthesis, RNAi-mediated expression knockdown assays were carried out in *T. wilfordii*. Three RNA interference (RNAi) fragments (314, 304, and 311 bp) designed according to the full-length coding sequence of *TwMYC2a* (Figure S6) were cloned into a pK7GWIWG2_II-RedRoot vector, containing a red fluorescent protein (RFP) gene, thereby generating three silencing constructs. After the introduction, the transgenic hairy roots lines (R1, R2, R3, and the control line, which introduced with the empty vector pK7GWIWG2_II-RedRoot) were confirmed by fluorescence identification and PCR analysis (Figure S7). We then conducted a qRT-PCR analysis of these transgenic lines. As shown in Figure 4A, the transcript levels of *TwMYC2a* and *TwMYC2b* in R1, R2, and R3 RNAi lines were significantly decreased by 55%~81% and 46%~73%, respectively, compared with the control line (EV). Among them, the expressions of *TwMYC2a* and *TwMYC2b* in the R1 hairy roots line were the lowest, 19% and 27% of the control line, respectively. Next, we chose the R1 line for further study. To evaluate how the expression of genes related to triptolide biosynthesis is influenced by TwMYC2a/b, expression levels of several key triptolide biosynthetic genes were analyzed in R1 and control (EV) hairy roots lines. As shown in Figure 4B, the expressions of downstream pathway genes *TwCPS* (*TwTPS7/9*) and *TwMS* (*TwTPS27a/b*) and upstream pathway genes *TwDXR* and *TwHMGR1* were significantly increased, by 2.66-, 2.12-, 2.21-, and 1.64-fold in R1 line, respectively, compared with the control line (EV). These results further confirmed the negative regulatory effect of TwMYC2a/b on *TwTPS27a/b* and other key pathway genes and also demonstrated that the miltiradiene synthase genes *TwTPS27a* and *TwTPS27b* were two target genes of TwMYC2a and TwMYC2b. 

### 2.5. RNAi of TwMYC2a/b Increases Triptolide Production in the Hairy Roots of T. wilfordii 

**Figure 5:**
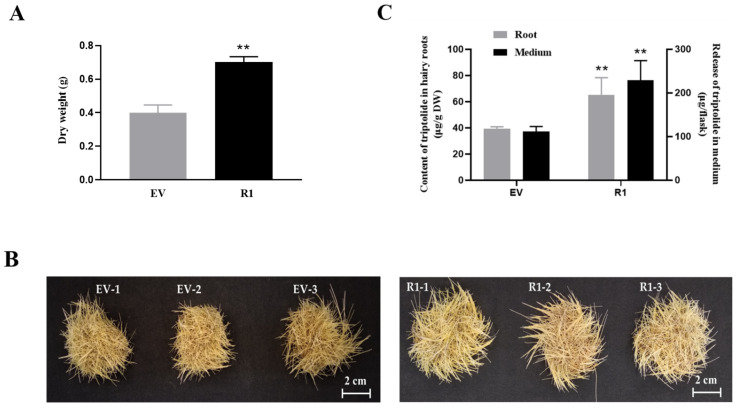
The biomass, phenotypes, and the production of triptolide in the control (EV) and *TwMYC2a/b* RNAi (R1) hairy roots lines. (**A**) The dry weight of the control (EV) and R1 hairy roots. (**B**) Phenotypes of the control (EV) and R1 hairy roots. Scale bars are 2 cm. (**C**) Triptolide content in hairy roots and the release content of triptolide in the medium in the control (EV) and R1. DW, dry weight. All the hairy roots were obtained after being cultured for 28 d. All data presented here are means ± SD of three biological replicates. Statistical significance was determined by Student’s *t*-test (** *p* < 0.01). These assays were repeated three times with similar results.

First, we measured the dry weight of the R1 and control (EV) hairy roots after being cultured for 28 d. As shown in Figure 5A, the biomass of the R1 line (0.70 g DW) was significantly higher than that of the control line (EV, 0.40 g DW) after being cultured for 28 d (1.76-fold). This finding was consistent with the phenotypes of the R1 and control (EV) hairy roots (Figure 5B).

Then, the accumulation of triptolide in R1 and control hairy roots was detected by High-Performance Liquid Chromatography (HPLC) (Figure 5C and Figure S8). The results showed that the content of triptolide in R1 hairy roots (65.38 DW μg/g) was significantly increased by 1.67-fold, compared with the control (EV, 39.20 DW μg/g). Besides, the release content of triptolide in the liquid medium of R1 (229.63 μg/flask) was also significantly higher than that of the control (EV, 111.49 μg/flask) (2.06-fold) (Figure 5C). These results indicated that TwMYC2a and TwMYC2b were negative regulators that affected the biosynthesis of triptolide in *T. wilfordii* hairy roots.

## 3. Discussion

MYC2, which belongs to the IIIe clade of the basic helix-loop-helix (bHLH) superfamily that is one of the largest group of transcription factors (TFs) in plants, is a core regulator involved in plant secondary metabolism [41]. In this study, two homologous MYC2 TF genes *TwMYC2a* and *TwMYC2b* were identified from *Tripterygium wilfordii* hairy roots by the 5′/3′-RACE method. Bioinformatics analysis showed that two domains were highly conserved in the TwMYC2a/b proteins and other MYC2 TFs: a bHLH-MYC superfamily domain at the N-terminal region and a basic helix-loop-helix leucine zipper domain at the C-terminal region. Besides, the 4aa-length peptide present in the TwMYC2a/b proteins (‘SDHS’ for TwMYC2a and ‘SEHS’ for TwMYC2b, Figure 1A), assumed to be the phosphorylation site, were only highly similar among MYC2 [41]. Phylogenetic analysis showed that TwMYC2a/b were grouped into the IIIe clade of the bHLH superfamily with other functional MYC2 proteins, such as CrMYC2 [37], AtMYC2 [27], TcMYC2a [42], NtMYC2a/b [43], HbMYC2 [44], HbMYC2b [45], and SmMYC2 [46] (Figure 1B). Thus, these findings indicate that TwMYC2a and TwMYC2b are two typical MYC2 TF and may play an important role in the regulation of secondary metabolism in *T. wilfordii*.

The bHLH TFs function by binding to the E-box (CANNTG) motif in their target gene promoters [31]. Our previous studies have isolated the promoters of key pathway genes *TwTPS27a* and *TwTPS27b* and silico analysis showed that five different types of E-box (CANNTG) motifs were found in their promoters [35]. It has been reported that in addition to the G-box (CACGTG) showing the strongest MYC2 binding capacity, the E-box (CACATG) and T/G-box (CACGTT) have stronger binding capacity than other E-box variants [27]. This implies that the E-box (CACATG) and T/G-box (CACGTT) present in the *TwTPS27a/b* promoters, as the potential MYC-binding sites, are most likely to be bound by TwMYC2a/b in *T. tripterygium*. In this study, to verify this hypothesis, yeast one-hybrid (Y1H) assays were conducted and demonstrated that these two potential MYC-binding sites, E-box (CACATG) and T/G-box (CACGTT), in the *TwTPS27a/b* promoters were indeed bound by TwMYC2a and TwMYC2b (Figure 2C). Besides, β-glucuronidase (GUS) transactivation assays were performed and further demonstrated that TwMYC2a/b negatively regulated the activity of the *TwTPS27a/b* promoters (Figure 3B,C). These results confirmed the interaction between the *TwTPS27a/b* promoters and TwMYC2a/b TFs. Then, the qRT-PCR analysis in the ectopic RNA interference-mediated knockdown experiments with hairy roots cultures of *T. wilfordii* proved that the miltiradiene synthase genes *TwTPS27a* and *TwTPS27b* were two target genes of TwMYC2a and TwMYC2b. Besides, these results will lay a foundation for studying the interaction between the key pathway genes of triptolide biosynthesis and the candidate transcription factors involved in the regulation of triptolide biosynthesis in *T. wilfordii*.

Recently, the release of the *T. wilfordii* genomic data [22] made it easier to analyze the potential MYC-binding sites in the triptolide biosynthetic gene promoters. In the genomic data of *T. wilfordii*, the downstream key pathway genes *TwTPS7/7v2* and *TwTPS9/9v2*, and the upstream key pathway genes *TwDXR* and *TwHMGR1*, were found in the chromosome of 21, 21, 18, and 7, respectively. The potential promoter sequences (2000 bp nucleotides upstream of their start codon ATG) of these biosynthetic genes were analyzed and showed that many E-box motifs present in their potential promoter sequences (Table S2). Notably, the E-box motifs ‘CACATG’ (demonstrated to be bound by TwMYC2a/b in Y1H assays, Figure 2C) or its reverse sequence ‘CATGTG’ were found in all these genes’ potential promoter sequences, and the T/G-box motif ‘CACGTT’ (demonstrated to be bound by TwMYC2a/b in Y1H assays, Figure 2C) was found in the potential promoter sequence of *TwHMGR1*. Therefore, the presence of many potential MYC-binding sites (various types of E-box motifs) in their potential promoters indicated that these pathway genes might be recognized and regulated by TwMYC2a/b. In this study, the relative expressions of these key pathway genes *TwCPS* (*TwTPS7/9*), *TwMS* (*TwTPS27a/b*), *TwDXR*, and *TwHMGR1* were significantly increased, by 2.66-, 2.12-, 2.21-, and 1.64-fold in RNAi-TwMYC2a/b hairy roots (R1 line), respectively, compared with the control line (EV) (Figure 4B). Compared with the control, the triptolide productions in the hairy roots and liquid medium of RNAi-TwMYC2a/b hairy roots (R1 line) were both significantly enhanced by 1.67- and 2.06-fold, respectively (Figure 5C). Therefore, these results further verified that these key pathway genes could be regulated by TwMYC2a/b, and TwMYC2a/b act negative roles in the regulation of triptolide biosynthesis.

In this study, we measured the dry weight of the R1 and control (EV) hairy roots after being cultured for 28 d and the results showed that the biomass of the R1 hairy roots line was significantly higher than that of the control line (1.76-fold) (Figure 5A). It is reported that AtMYC2 inhibits primary root growth in *A. thaliana* [53,54]. Similarly, AaMYC2 from *Artemisia annua* and MdMYC2 from *Malus domestica* exhibit the same function as AtMYC2 in *Arabidopsis* seedlings [55,56]. Therefore, we speculate that TwMYC2a/b also has the potential effect on inhibiting root growth in *T. wilfordii*. This hypothesis may explain the increased dry weight of the RNAi-*TwMYC2a/b* hairy roots line (R1 line). However, SmMYC2b from *S. miltiorrhiza* exerts opposite functions in inhibiting root growth [57]. Notably, the phylogenetic analysis showed that SmMYC2b was grouped into the IIId clade of the bHLH superfamily with other functional JAM proteins, such as AtJAM1, AtJAM2, and AtJAM3 from *A. thaliana* [25,58]. Besides, JAM is antagonistic to MYC2 and negatively regulates the inhibition of root growth [59,60]. Therefore, it is easy to understand why SmMYC2b, as a member of JAM TF, plays the opposite role in inhibiting root growth, rather than showing the same inhibitory effect as the real MYC2 TF. In addition, the triptolide accumulation in the hairy roots and liquid medium of RNAi-TwMYC2a/b hairy roots (R1 line) were both significantly higher than that of controls (Figure 5C). Given this, RNAi-TwMYC2a/b hairy roots line (R1 line) with rapid growth and high content of triptolide will provide an important candidate resource for the metabolic engineering of triptolide.

In the regulation of plant secondary metabolism, many key enzyme gene promoters can be recognized by positive or negative regulators, thereby increasing or decreasing the content of secondary metabolites. For example, the promoter of *SmKSL*, a key tanshinone biosynthetic gene encoding miltiradiene synthase in *S miltiorrhiza*, containing GCC-box and E-box motifs, can be recognized by positive regulators SmERF6 [61] and SmERF128 [62], and negative regulator SmbHLH3 [63], thereby up-regulating and down-regulating the biosynthesis of tanshinone. Similarly, in *T. wilfordii*, the promoters of miltiradiene synthase genes *TwTPS27a* and *TwTPS27b* containing TGACG-motif, E-box, and T/G-box elements also can be regulated by positive regulator TwTGA1 upregulating triptolide biosynthesis [35,64] and negative regulators TwMYC2a and TwMYC2b reported here. These findings indicate that the regulation of triptolide biosynthesis is complex and more potential transcription factors can be involved in the biosynthesis of triptolide, which needs further study.

## 4. Materials and Methods

### 4.1. Plant Materials, Growth Conditions, RNA Isolation, and cDNA Synthesis

The plant of *Tripterygium wilfordii* was grown beside the grape trellis of Shaanxi Research Center of Biopesticide Engineering & Technology, Northwest A&F University, YangLing, China. The hairy roots and sterile seedlings of *T. wilfordii* were maintained and subcultured in our laboratory [9]. Tobacco (*Nicotiana benthamiana*) plants for agroinfiltration were grown under 16 h day/8 h night (24 ± 1 °C) conditions in a plant growth chamber for five or six weeks. The Y1H Gold yeast cells were used for yeast one-hybrid (Y1H) assays. The extraction of total RNA was conducted according to the instruction manuals of the Plant Total RNA Extraction Kit (DNA-free) (Biospin, Shanghai, China). The first-strand cDNA synthesis for gene cloning and expression was performed according to the manufacturer’s protocol of PrimeScript™ II 1st Strand cDNA Synthesis Kit (TaKaRa, Dalian, China). The first-strand cDNA synthesis for quantitative real-time PCR (qRT-PCR) was performed according to the manufacturer’s instruction of PrimeScript™ RT reagent Kit with gDNA Eraser (TaKaRa, Dalian, China).

### 4.2. Isolation and Bioinformatics Analyses of the TwMYC2a and TwMYC2b

The Basic Local Alignment Search Tool (BLAST) search was conducted for homolog searching, using the tBLASTn (protein sequence searched against translated nucleotide sequences) mode, from *T. wilfordii* transcriptome library (SRX472292), and the functional protein sequence of AtMYC2 (also called RD22BP1, GenBank accession NO. BAA25078) from *Arabidopsis thaliana* as the query sequence. One candidate gene, *comp41180_c0_seq5*, was screened with the highest score (bits) (Figure S1). To clone the 5′/3′-untranslated region (5′/3′-UTR) of this candidate gene, 5′/3′-rapid amplification of cDNA ends (5′/3′-RACE) assays were conducted according to the protocol of SMARTer RACE 5′/3′ Kit (Clontech, CA, USA). Specific primers for the first round 5′/3′-RACE polymerase chain reaction (PCR) (MYC2-5’-R1-1066 and MYC2-3’-F1-1192, Table S3) and second round 5′/3′-RACE PCR (MYC2-5’-R2-795 and MYC2-3’-F2-1578, Table S3) were designed according to the candidate gene sequence. After the second round 5′/3′-RACE PCR cloning, the PCR products were purified and then introduced into the pMD19-T clone vector by TA cloning procedure. After sequencing and alignment, the 5′/3′-UTR of the candidate gene was confirmed. According to the 5′/3′-UTR and the candidate gene sequence, the primers for cloning the full-length CDS of the candidate gene were designed (Table S3). Finally, through sequence alignment, two homologous full-length cDNA of the candidate gene were obtained and named *TwMYC2a* and *TwMYC2b*, respectively.

The nucleotide sequence and open reading frame (ORF) of *TwMYC2a/b* were analyzed by ORF Finder (https://www.ncbi.nlm.nih.gov/orffinder/ (accessed on 1 July 2016)). The theoretical isoelectric point (pI) and molecular weight (Mw) of the TwMYC2a/b protein sequences were analyzed by the Compute pI/Mw tool (https://web.expasy.org/compute_pi/ (accessed on 1 July 2016)). The conserved domains of these two protein sequences were predicted using NCBI’s conserved domain database (https://www.ncbi.nlm.nih.gov/Structure/cdd/wrpsb.cgi (accessed on 1 July 2016)). Using DNAMAN version 9, the protein sequences of TwMYC2a/b were compared with other homologous plant MYC2 proteins. The phylogenetic tree of TwMYC2a/b with other functional MYC2 protein sequences was constructed by the neighbor-joining method using the MEGA7 software with 1000 bootstraps run.

### 4.3. Subcellular Localization of TwMYC2a and TwMYC2b

The coding sequences of *TwMYC2a* and *TwMYC2b* removing the stop codon with *Eco*R I and *Kpn* I restriction sites were separately cloned into a modified vector pCAMBIA1302 (35S-eGFP) to generate the recombinant vectors 35S-TwMYC2a-eGFP and 35S-TwMYC2b-eGFP. The 35S-eGFP was used as the control vector. All of these vectors were separately transformed into *Agrobacterium tumefaciens* GV3101 (pSoup-p19) and infiltrated into tobacco (*Nicotiana benthamiana*) leaves using *Agrobacterium*-mediated transient transformation. After 48 h of cultivation, the fluorescence in the transformed tobacco leaves was observed using Leica TCS SP8 MP confocal microscope (Leica, Bensheim, Germany). All the primers used for the construction of subcellular localization vectors are listed in Table S3.

### 4.4. Quantitative Real-Time PCR (qRT-PCR) Analysis

The qRT-PCR assays for gene expression analysis were performed according to our previous method [9]. The relative gene expression was quantified by using the 2^−ΔΔCt^ method [65]. Elongation factor 1 α (EF1α) was used as the internal standard for gene expression normalization. The qRT-PCR primers of *TwMYC2a* and *TwMYC2b* were designed in the 3′-untranslated region (3′-UTR) of *TwMYC2a/b* (Figure S9). All the primers used for qRT-PCR analysis are listed in Table S3.

### 4.5. Yeast One-Hybrid (Y1H) Assay

To detect whether TwMYC2a/b bound to the E-box (CACATG) and T/G-box (CACGTT) of *TwTPS27a/b* promoters in yeast, the Y1H assays were performed according to the manufacturer’s instruction (Clontech, CA, USA). The triple repeated sequence ‘ATTCACATGTAA’ and ‘CATCACGTTAGA’ (three bases of flanking sequence together with the core elements CACATG/CACGTT) were artificially synthesized and separately cloned into pAbAi (Clontech, CA, USA) to generate the bait vectors. The coding sequences of *TwMYC2a* and *TwMYC2b* were amplified by PCR and separately fused to the yeast GAL4 activation domain (GAL4 AD) of pGADT7-Rec (Clontech, CA, USA) to generate the prey vectors. Then the bait vectors were linearized and integrated into the genome of Y1H Gold yeast strain, selected on SD/-Ura agar medium plate, which was cultured at 30 °C for 3 d. Next, the prey vectors and the blank pGADT7-Rec (AD, used as a control), were introduced into the yeast cells previously transformed with the bait vectors, respectively. The concentration of positively transformed yeast cells that harbor different combinations of bait and prey vectors were adjusted to OD600 ≈ 1.0 and serially diluted 1/10, 1/100, 1/1000 in sterile ddH_2_O. The serial dilution of transformed yeast cells was cultured on SD/−Leu medium plates with an optimal concentration of aureobasidin A (AbA) to examine any protein-DNA interactions. Plates were incubated at 30 °C for 3 d. All the primers used for the construction of the bait and prey vectors are listed in Table S3.

### 4.6. GUS Transactivation Assay

To verify whether TwMYC2a/b could regulate the *TwTPS27a/b* promoters, GUS transactivation assays were performed in tobacco leaves. The full-length CDSs of *TwMYC2a/b* were separately cloned into a pJCV53 vector (Invitrogen, CA, USA) containing a red fluorescent protein (RFP) gene, using the Gateway cloning system (Invitrogen, CA, USA), thereby generating the effector vectors, 35S::TwMYC2a and 35S::TwMYC2b. The empty vector (EV) was used as a negative control. The promoters of *TwTPS27a* (1494 bp upstream of the start codon) and *TwTPS27b* (1862 bp upstream of the start codon) were cloned into the pBI121 vector, generating the reporter vectors, 27aP::GUS and 27bP::GUS, which had been previously constructed [35]. All of these constructs were separately transformed into *Agrobacterium tumefaciens* GV3101 (pSoup-p19). The bacterial cultures harboring the effectors (35S::TwMYC2a/b or EV) and reporters (27aP::GUS or 27bP::GUS) were resuspended in 2-(N-morpholino)ethanesulfonic acid (MES) buffer to OD_600_ of 1.2 and 0.8, respectively, and mixed in the ratio of 1:1 (v:v) before infiltration. Each mixed bacterial suspension was infiltrated into the left and right sides of the midrib in the same leaf, respectively. The GUS fluorimetric activity and GUS staining of these infiltrated tobacco leaves were analyzed until 72 h after infiltration. These assays were performed as described in our previous study [35] and repeated at least three times with similar results. All the primers used for the construction of effector and reporter vectors are listed in Table S3.

### 4.7. Construction of RNAi Vector and Generation of TwMYC2a/b Transgenic RNAi Hairy Roots

According to the full-length coding sequence of *TwMYC2a*, three RNA interference (RNAi) fragments, 314, 304, and 311 bp, were designed (Figure S6). These three RNAi fragments were then separately cloned into a pK7GWIWG2_II-RedRoot vector (Invitrogen, CA, USA) containing a red fluorescent protein (RFP) gene, using the Gateway cloning system, thereby generating three RNAi vectors, named pKR-RNAi-1, pKR-RNAi-2, and pKR-RNAi-3, respectively. All these constructs were then introduced into *Agrobacterium rhizogenes* ATCC15834 via a freeze-thaw method. All the primers used for the construction of the RNAi vectors are listed in Table S3.

The induction of transgenic hairy roots was performed as described in our previous study [9]. The hairy roots line harboring the empty vector (pK7GWIWG2_II-RedRoot) was used as control.

### 4.8. Transformant Selection and Transcript Analysis

The positive transgenic hairy roots lines were confirmed by fluorescence identification and PCR analyses as described previously [9]. Genomic DNA was extracted from the transgenic hairy roots lines according to the instruction manuals of the Plant Genomic DNA Extraction Kit (Biospin, Shanghai, China). All the primers used for the identification of transgenic hairy roots lines are listed in Table S3.

Transcript abundances of *TwMYC2a/b* and several key pathway genes were analyzed by qRT-PCR. All the primers used for qRT-PCR analysis are listed in Table S3.

### 4.9. High-Performance Liquid Chromatography (HPLC) Analysis

For the quantification of triptolide, the 28-day-old transgenic hairy roots were dried at 45 °C to a constant weight in an oven and then ground to powder. The sample powder (100 mg) was extracted with absolute ethanol (1 mL) under ultrasonic treatment for 30 min (repeated three times). The resulting mixture was centrifuged at 12, 857 g for 5 min. The extractive supernatant was filtered through a 0.22 μm organic membrane filter. After the sample was evaporated to dryness, it was dissolved in 1 mL of acetonitrile and filtered through a 0.22 μm organic membrane filter again. All the samples were analyzed by HPLC. The procedures of HPLC analysis of triptolide were the same as described previously [66].

### 4.10. Statistical Analysis

All data were presented as means ± SD of three biological replicates. Statistical analyses were carried out using the SPSS23.0 (SPSS, IBM, USA) software. The statistically significant difference was determined by Student’s *t*-test (*, *p* < 0.05; **, *p* < 0.01).

## 5. Conclusions

In the present study, two MYC2 transcription factors, TwMYC2a and TwMYC2b, were identified as negative rather than positive regulators involved in the regulation of triptolide biosynthesis in the hairy roots of *T. wilfordii*. These findings here extend our understanding of the transcriptional regulation of triptolide biosynthesis and also provide new light on metabolic engineering of triptolide in the future.

## Data Availability

The data that support the findings of this study are available from the corresponding author, [X.Z.], upon reasonable request.

## Supplementary Materials

The following are available online at https://www.mdpi.com/article/10.3390/plants10040679/s1, Table S1: Full names of several functional pathway genes in triptolide biosynthesis, Table S2: The numbers of various E-box motifs in the protential promoter sequences of *TwTPS7*, *TwTPS9*, *TwDXR*, and *TwHMGR1* (2000 bp nucleotides upstream of their start condon ATG), Table S3: Primers used in this study, Figure S1: Results of tBLASTn search in *T. wilfordii* transcriptome library (SRX472292), Figure S2: Identification of the PCR products for the second round of the 5′/3′-rapid amplification of cDNA ends (5′/3′-RACE) (A) and the full-length coding sequence (CDS) (B) of candidate genes (*TwMYC2a/b*), Figure S3: Sequence alignment between the 5′/3′-RACE sequences and the candidate gene sequence, Figure S4: Sequence alignment between the 5′/3′-RACE sequences and the partial CDSs of *TwMYC2a* and *TwMYC2b*, Figure S5: Alignment the full-length CDSs and protein sequences between *TwMYC2a* and *TwMYC2b*, Figure S6: Positions of the three RNAi fragments exist in the cDNA of *TwMYC2a*, Figure S7: Identification of RNAi transgenic hairy roots lines, Figure S8: Chromatograms of the standard Triptolide and samples (three biological replicates of EV and R1 hairy roots line), Figure S9: Primers were designed in the 3’-untranslated region (3’-UTR) of *TwMYC2a/b* for qRT-PCR assays of *TwMYC2a/b*.
