# Supplementary material for: MYC2 Transcription Factors TwMYC2a and TwMYC2b Negatively Regulate Triptolide Biosynthesis in Tripterygium wilfordii Hairy Roots
Source: Plants (Basel). 2021 Apr 1;10(4):679. doi: 10.3390/plants10040679 (PMC8067133; doi:10.3390/plants10040679)
Supplement: Supplementary file 1 [file plants-10-00679-s001.pdf]

Supplementary materials

Table S1. Full names of several functional pathway genes in triptolide biosynthesis

Genes	Full names
TwHMGR1	3-hydroxy-3-methylglutaryl- coenzyme A reductase gene
TwHMGS	3-hydroxy-3-methylglutaryl-coenzyme A synthase gene
TwDXR	1-deoxy-d-xylulose-5-phosphate reductoisomerase gene
TwDXS1/2	1-deoxy-d-xylulose 5-phosphate synthase genes
TwHDR	4-hydroxy-3-methylbut-2-enyl diphosphate reductase gene
TwIDI	isopentenyl pyrophosphate isomerase gene
TwGGPPS1/4/8	geranylgeranyl diphosphate synthase genes
TwTPS7/7v2/9/9v2	*normal*-copalyl diphosphate synthase genes
TwTPS27/27v2	miltiradiene synthase genes
TwCYP728B70	cytochrome P450 gene

Table S2. The numbers of various E-box motifs in the protentical promoter sequences of *TwTPS7*, *TwTPS9*, *TwDXR*, and *TwHMGR1* (2000 bp nucleotides upstream of their start condon ATG).

Name	E-box Sequences	Numbers of E-box			
		TwTPS7	*TwTPS9*	*TwDXR*	*TwHMGR1*
E1	CAGATG/CATCTG	1	0	1	0
**E2**	**CACATG/CATGTG**	**3**	**2**	**2**	**2**
E3	CAAATG/CATTTG	2	4	2	3
**E4**	**CACGTT/AACGTG**	**0**	**0**	**0**	**1**
E5	CAATTG	1	2	2	0
E6	CATATG	1	3	0	2
E7	CAACTG/CAGTTG	1	0	1	1
E8	CAAGTG/CACTTG	1	1	1	1
E9	CAGGTG/CACCTG	0	1	1	0
E10	CAGCTG	0	0	0	1
G-box	CACGTG	0	0	0	2
Total		10	13	10	13

Note: These patyway genes *TwTPS7*, *TwTPS9*, *TwDXR*, and *TwHMGR1* are located in the chromosome of 21, 21, 18, and 7, respectively. The 2000 bp nucleotides upstream of their start condon ATG in their genomic sequences were analyzed as the potential promoter sequence.

Table S3. Primers used in this study

Primers	Sequence (5' to 3')
5'/3'-RACE PCR	
MYC2-5'-R1-1066	GACGAAGGTGATTTGGGATATGGACTC
MYC2-5'-R2-795	GGCACCACCATTACTGAAATTGAACA
MYC2-3'-F1-1192	TTTGGGGAGAGTAAGAGGACTGCTACCA
MYC2-3'-F2-1578	TGCTGTTGTTCCTAATGTGTCCAAGATG
Full-length CDS PCR	
MYC2a-F	ATGACGGACTACCGGCTCCCAGTAT
MYC2a-R	TTACCGAGCACCAACCCTGGATTGT
MYC2b-F	CCGGTGCATGAAATGACGGACTA
MYC2b-R	GTCCTATCGAGAATCACCAATCCTGG
Construction of the subcellular localization vectors	
MYC2a-sub-F	AGATTTATAAAAAAAAAAAAGAATTCATGACGGACTACCGGCTCC
MYC2a-sub-R	TCCTCGCCCTTGCTCACCATGGTACCCCGAGCACCAACCCTGG
MYC2b-sub-F	AGATTTATAAAAAAAAAAAAGAATTCATGACGGACTACCGGCTAC
MYC2b-sub-R	TCCTCGCCCTTGCTCACCATGGTACCTCGAGAATCACCAATCCTGG
qRT-PCR	
EF1α-F	CCAAGGGTGAAAGCAAGGAGAGC
EF1α-R	CACTGGTGGTTTTGAGGCTGGTATCT
MYC2a-q-F	CTGAACTAAGGGAAAATAGGATCGG
MYC2a-q-R	TAACAGAAGGATGGCCAATACAAAA
MYC2b-q-F	AGCTGAACTAAGGGAAAATCGC
MYC2b-q-R	GGAACAGTGAAGCAGGATTACC
TwTPS27a/b-q-F	ATGAATCAACGGCCCTTGACT
TwTPS27a/b-q-R	TCCTAATCGCTGCATCGACTC
TwTPS7/9-q-F	GCTAGAAAAAGACGATTCCGAGC
TwTPS7/9-q-R	ATAGCTTGCAAGAATGGCGAATC
TwHMGR1-q-F	CATGTTTGAACCTGCTTGGGG
TwHMGR1-q-R	GGCTTTTCACAAGCTGTCCAG
TwDXR-q-F	TCAAGGATTGCCAGAGGG
TwDXR-q-R	ATGAATGATAGACTGCGGATG
Construction of bait vectors	
3×E2-AbAi-HindIII-F	AGCTTATTCACATGTAAATTCACATGTAAATTCACATGTAAC
3×E2-AbAi-XhoI-R	TCGAGTTACATGTGAATTTACATGTGAATTTACATGTGAATA
3×E4-AbAi-HindIII-F	AGCTTCATCACGTTAGACATCACGTTAGACATCACGTTAGAC
3×E4-AbAi-XhoI-R	TCGAGTCTAACGTGATGTCTAACGTGATGTCTAACGTGATGA
Construction of prey vectors	
MYC2a-AD-F	GCCATGGAGGCCAGTGAATTCATGACGGACTACCGGCTCC
MYC2a-AD-R	ACGATTCATCTGCAGCTCGAGTTACCGAGCACCAACCCTGG
MYC2b-AD-F	GCCATGGAGGCCAGTGAATTCATGACGGACTACCGGCTAC
MYC2b-AD-R	ACGATTCATCTGCAGCTCGAGCTATCGAGAATCACCAATCCTGG
Construction of effector vectors (overexpression vectors)	
MYC2a-OE-F	GGGGACAAGTTTGTACAAAAAAGCAGGCTTCACCATGACGGACTACCGGCTCCCAGTAT
MYC2a-OE-R	GGGGACCACTTTGTACAAGAAAGCTGGGTCTTACCGAGCACCAACCCTGGATTGT
MYC2b-OE-F	GGGGACAAGTTTGTACAAAAAAGCAGGCTTCACCCCGGTGCATGAAATGA

	CGGACTA
MYC2b-OE-R	GGGGACCACTTTGTACAAGAAAGCTGGGTCGTCCTATCGAGAATCACCAATCCTGG
Construction of reporter vectors (promoter::GUS vectors)	
27aP-F	GACCATGATTACGCCAAGCTTGTACTGAATAAATAAATTAAATTGCTACAGTTATCG
27aP-R	ACCACCCGGGGATCCTCTAGAAATTCCCAGAAGAAAGGTGTGATTTT
27bP-F	GACCATGATTACGCCAAGCTTTGGGCCCTCTTTATTGAAAACAAAAAT
27bP-R	ACCACCCGGGGATCCTCTAGAAATTCCCAGAAGAAAGGTGTGATTTT
Construction of RNAi vectors	
MYC2-RNAi-1-F	GGGGACAAGTTTGTACAAAAAAGCAGGCTTCTATGCCCTACGTGCTGTT
MYC2-RNAi-1-R	GGGGACCACTTTGTACAAGAAAGCTGGGTTGGATGGTTCCTTTTGCTAC
MYC2-RNAi-2-F	GGGGACAAGTTTGTACAAAAAAGCAGGCTCTGGGTCATGTTTTCGGGTT
MYC2-RNAi-2-R	GGGGACCACTTTGTACAAGAAAGCTGGGTTGGTGTGGCTGCTGCTATTG
MYC2-RNAi-3-F	GGGGACAAGTTTGTACAAAAAAGCAGGCTTCTCAGCCTTCTGTCACTTTCAA
MYC2-RNAi-3-R	GGGGACCACTTTGTACAAGAAAGCTGGGTCACTCAGTATCAGTCACCTCTTC

Note: The underlined sequences represent homologous recombination sequence. The start/stop codons are marked with red color. The E-box sequences are marked with green color. The restriction site sequneces are marked with purple color.

```
Query=
         (623 letters)

Database: D:\BioEdit_7.0.9\database\Unigene.fa
           67,931 sequences; 47,438,532 total letters



                                                                 Score    E
Sequences producing significant alignments:                      (bits) Value

comp41180_c0_seq5                                                   656   0.0
comp40130_c0_seq4                                                   243   2e-064
comp39963_c0_seq1                                                   140   2e-033
comp20233_c0_seq1                                                   104   2e-022
comp32866_c0_seq1                                                   102   9e-022
comp236553_c0_seq1                                                   94   3e-019
comp34060_c0_seq2                                                    93   6e-019
comp31125_c0_seq1                                                    86   5e-017
comp77870_c0_seq1                                                    78   2e-014
comp38681_c1_seq5                                                    77   4e-014
comp30553_c0_seq1                                                    74   3e-013
comp23259_c0_seq1                                                    70   4e-012
comp159021_c0_seq1                                                   70   4e-012
comp32172_c0_seq2                                                    68   2e-011
comp380288_c0_seq1                                                   67   3e-011
comp10848_c0_seq1                                                    67   4e-011
comp148314_c0_seq1                                                   62   8e-010
comp222427_c0_seq1                                                   62   8e-010
comp33182_c0_seq1                                                    62   1e-009
comp32780_c0_seq2                                                    55   1e-007
```

Figure S1. Results of tBLASTn search in *T. wilfordii* transcriptome library (SRX472292). The project No. of *T. wilfordii* transcriptome library used in this study is SRX472292. The query sequense is the protein sequence of AtMYC2 (also called BAA25078, 623aa).

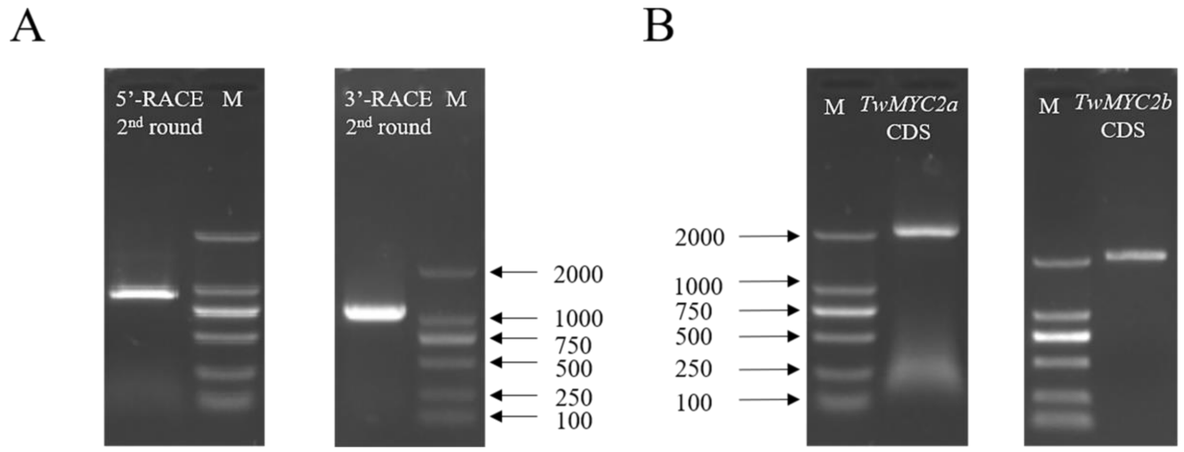

Figure S2. Identification of the PCR products for the second round of the 5'/3'-rapid amplification of cDNA ends (5'/3'-RACE) (A) and the full-length coding sequence (CDS) (B) of candidate genes (*TwMYC2a*/*b*). M: DL2000 Marker. The primers for cloning the full-length CDS of *TwMYC2a* were MYC2a-F and MYC2a-R (Table S2), and the MYC2b-F and MYC2b-R (Table S2) for *TwMYC2b*.

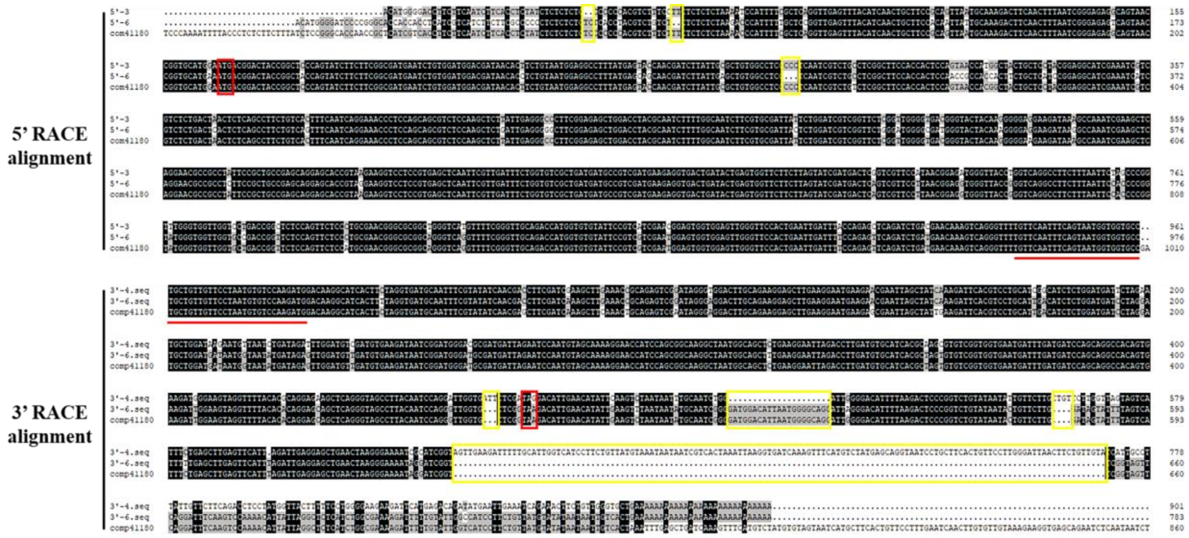

Figure S3. Sequence alignment between the 5'/3'-RACE sequences and the candidate gene sequence. The primers used for 2nd round 5'/3'-RACE PCR are marked with a sigle red line. The start codon (ATG) and stop codon (TAG/TAA) are marked with a red box. The gaps in sequence alignment are marked with a yellow box.

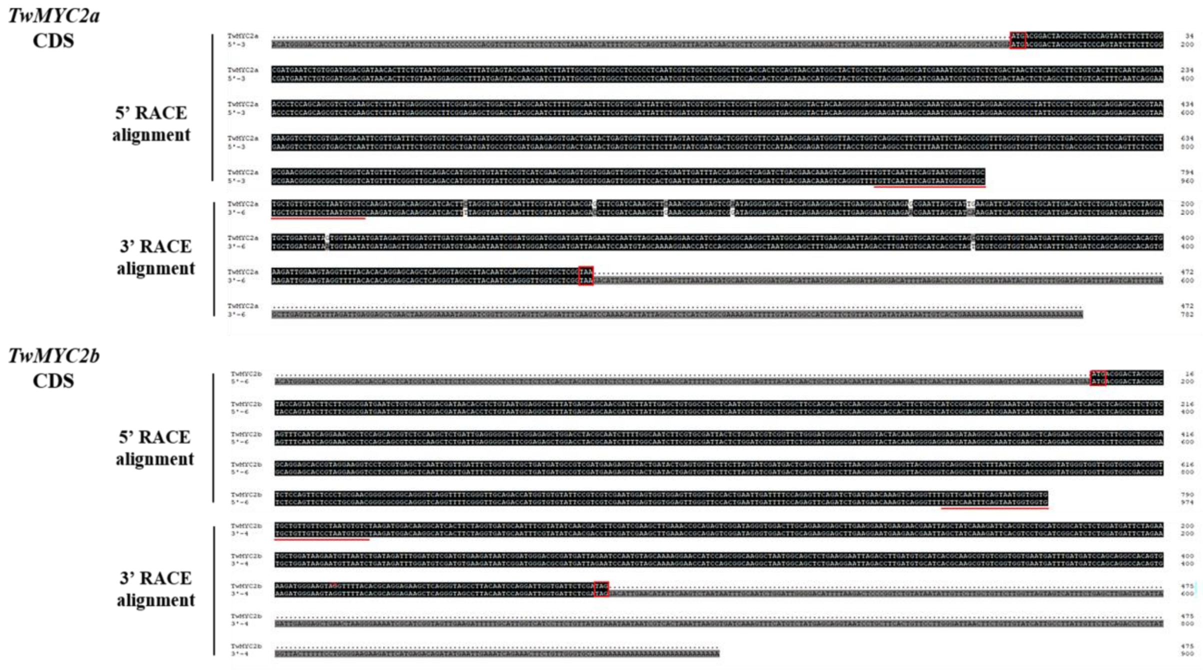

Figure S4. Sequence alignment between the 5'/3'-RACE sequences and the partial CDSs of *TwMYC2a* and *TwMYC2b*. The primers used for 2nd round 5'/3'-RACE PCR are marked with a sigle red line. The start codon (ATG) and stop codon (TAA/TAG) are marked with a red box.

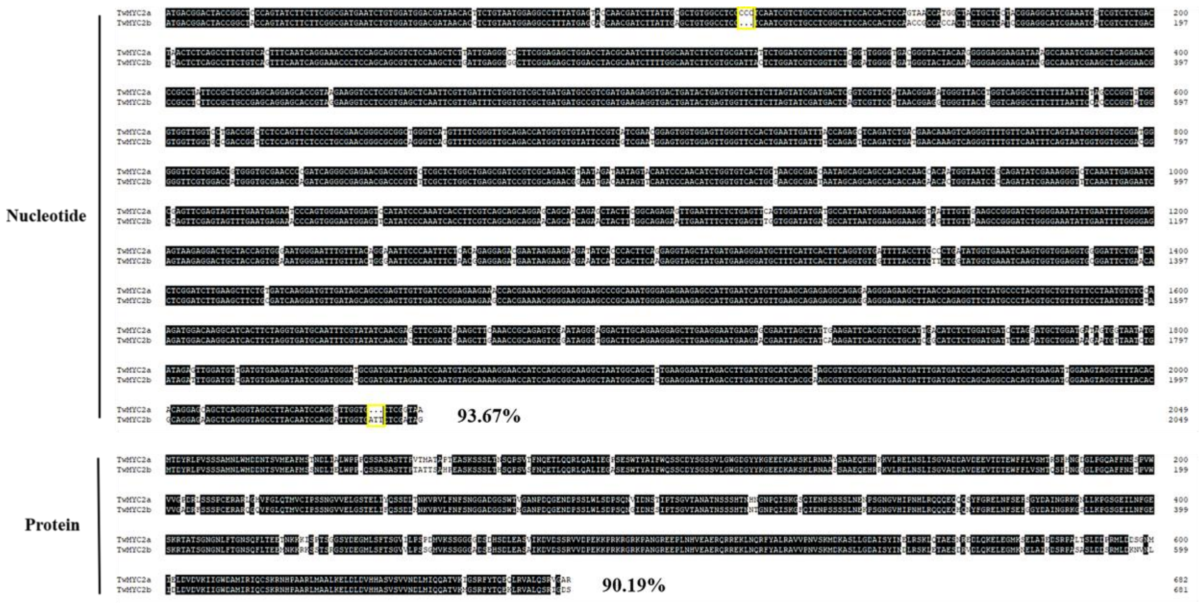

Figure S5. Alignment the full-length CDSs and protein sequences between *TwMYC2a* and *TwMYC2b*. Yellow boxes indicate the gaps of nucleotide sequences between *TwMYC2a* and *TwMYC2b* .

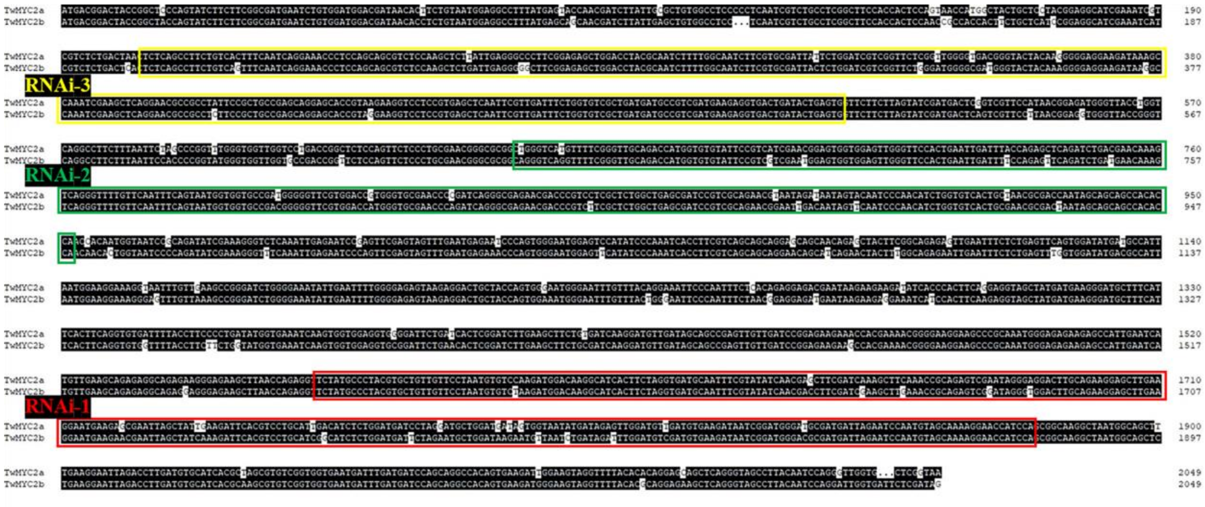

Figure S6. Positions of the three RNAi fragments exist in the cDNA of *TwMYC2a*. RNAi-1 (314 bp) fragment is marked with the red boxes. RNAi-2 (304 bp) fragment is marked with the green boxes. RNAi-3 (311 bp) fragment is marked with the yellow boxes.

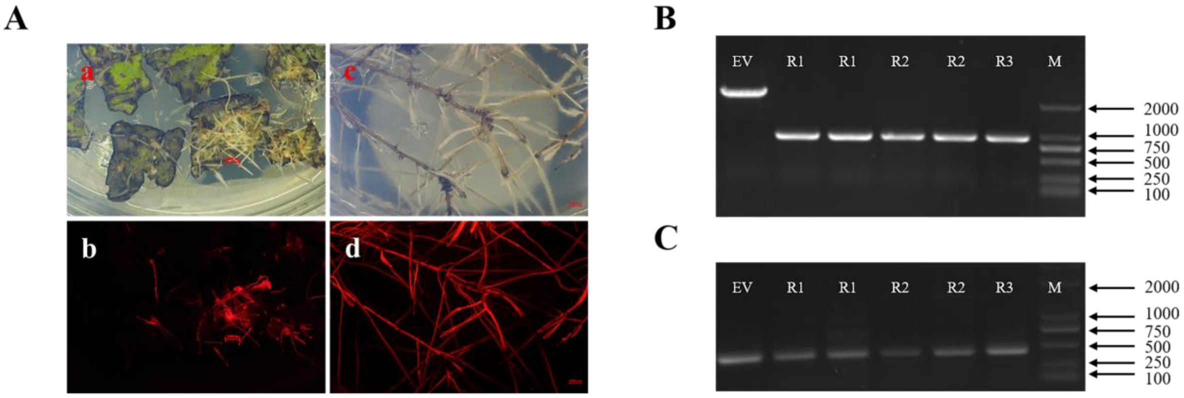

Figure S7. Identification of RNAi transgenic hairy roots lines. (A) DsRed-positive hairy roots under fluorescence microscopy with white light, (a) and (c), and exciting light, (b) and (d). The hairy root lines were acquired from the transformation of leaf explants of the *T. wilfordii* sterile plantlets (B) and (C): PCR analysis further confirmed the positive transgenic hairy root lines that has been identified by fluorescence identification. M: DL 2000 DNA marker. EV: control hairy root; R1, R2, and R3: RNAi transgenic hairy roots containting the conresponding RNAi vector, pKR-RNAi-1, pKR-RNAi-2, and pKR-RNAi-3, respectively, and have been identified by fluorescence identification. The sequencing primers pKR-F: 5'-CACTATCCTTCGCAAGACCCT-3' and pKR-R: 5'-CTCTGGAGTGAATACCACGACGAT-3' were used to validate the transformed hairy roots in (B). The rolB gene in RNAi transgenic hairy roots was verified by PCR with primers rolB-F：5'-GCTCTTGCAGTGCTAGATTT-3' and rolB-R：5'-GAAGGTGCAAGCTACCTCTC-3' (C).

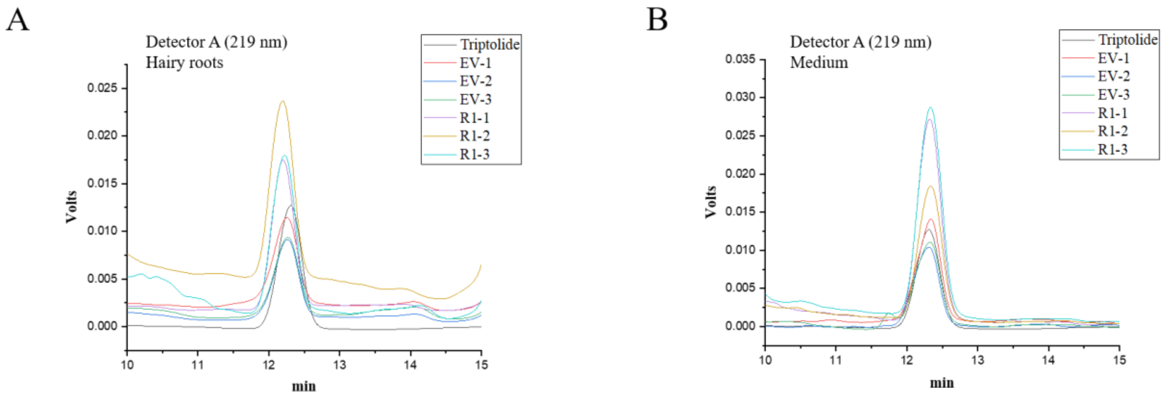

Figure S8. Chromatograms of the standard Triptolide and samples (three biological replicates of EV and R1 hairy roots line). (A) The detection of triptolide in hairy roots of EV and R1 lines by HPLC. (B) The detection of triptolide in the medium of EV and R1 hairy roots lines by HPLC.

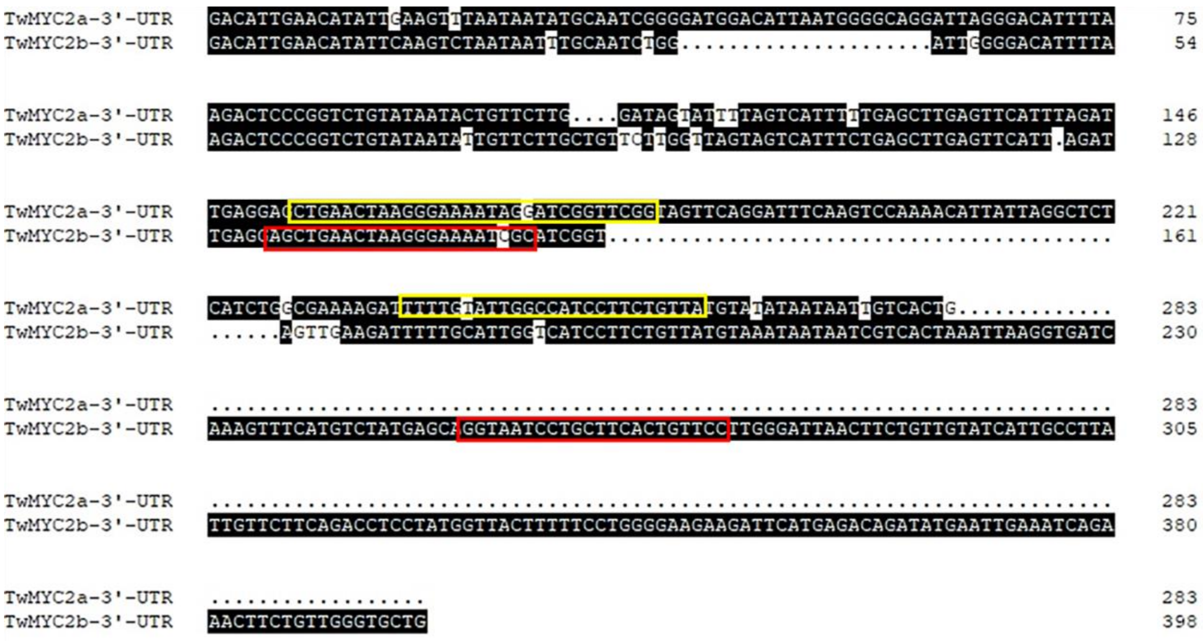

Figure S9. Primers were designed in the 3'-untranslated region (3'-UTR) of *TwMYC2a/b* for qRT-PCR assays of *TwMYC2a/b*.
